# Imaging Findings in Dogs and Cats With Presumptive Sclerosing Encapsulating Peritonitis

**DOI:** 10.3389/fvets.2022.891492

**Published:** 2022-06-09

**Authors:** Bérengère C. H. Gremillet, Charles Porsmoguer, Géraldine Bolen, Frédéric Billen, Stéphanie Noël, Flore Brutinel, Valeria Busoni

**Affiliations:** Département des Animaux de Compagnie, Clinique Vétérinaire Universitaire, Université de Liège, Liège, Belgium

**Keywords:** feline, canine, diagnostic imaging, sclerosing encapsulating peritonitis, ultrasound, radiograph (X-ray), computed tomography

## Abstract

This retrospective case series describes imaging findings in seven dogs and two cats with a presumptive diagnosis of sclerosing encapsulating peritonitis (SEP) between 2014 and 2021. Peritoneal effusion was present in all animal patients. Sonographically, echogenic fluid with or without echogenic intraperitoneal septations, gathered or corrugated bowel loops, and abdominal lymphadenomegaly were suggesting an inflammatory process and the presence of adhesions. Gathering of the bowel with abdominal distension and/or signs of intestinal obstruction were major findings on radiographs. Abdominal fat stranding was an additional finding in animals undergoing a CT examination. Previous surgery, pregnancy, and the presence of a perforating foreign body were potential predisposing causes in 4/9 animals. Peritonitis was septic in 4/9 animals. As SEP is a rare condition but life threatening, this detailed description of imaging findings in a short case series can be useful for a presumptive diagnosis and surgical planning.

## Introduction

Sclerosing encapsulating peritonitis (SEP), also referred as “chronica fibrosa encapsulata,” “icing sugar bowel” or “fibroplastic peritonitis” in the literature, is defined as a chronic inflammatory condition often of unknown etiology in which the small intestines are encased in a dense fibrocollagenous membrane (1, 2). It is believed that this pathology results from low grade or subclinical peritonitis that eventually progressed to multiple adhesions and membrane formation, with sclerosing granulation or fibrous tissue encapsulation, and sometimes distortion of the viscera (1, 2).

This pathological entity is uncommon in dogs and rare in cats (3–16). Only few cases are published and beside three case series published in the 90's (3–6), the most recent cases enriching the veterinary literature are single case reports (8–16). In humans, SEP most commonly manifests as recurrent acute, subacute or chronic intestinal obstruction. Clinical presentation of SEP in animals is variable and rather nonspecific: most cases present with chronic peritoneal effusion with or without digestive signs, including vomiting, diarrhea, anorexia, and abdominal pain (4–7, 9–16). Some animals diagnosed with SEP are reported with a history of abdominal foreign bodies or chronic bacterial infections but most of the time the disease seems to be idiopathic (17). Therefore the designation primary idiopathic SEP as opposed to secondary SEP has been suggested (1, 2).

Because imaging is a routine part of the work-up of cases with abdominal symptoms, the objective of this retrospective study is to provide a detailed description of the most common imaging findings of SEP determined from a case series of dogs and cats having a final diagnosis of SEP at surgery.

## Materials and Methods

Case records from the Small Animal Veterinary Clinic of the University of Liège from 2014 to 2021 were reviewed. Animals with a final diagnosis of SEP based on macroscopic findings and/or histopathological analysis of biopsies at surgery were included in the retrospective study and their data (signalment, history, clinical findings, imaging reports and diagnostic images) reviewed. All included animals had undergone a comprehensive clinical examination and a diagnostic imaging investigation, including at least abdominal ultrasonography, with or without abdominal radiography or computed tomography (CT).

Ultrasound examination was performed in dorsal recumbency on awake animals using a fixed ultrasound machine equipped with high frequency (7.5–13 MHz) linear and/or curvilinear probes^1^, ^2^, ^3^.

Radiographs included at least two orthogonal views: a latero-lateral projection and a ventro-dorsal projection. All radiographs were performed on awake animals maintained by human operators using a computed or direct radiography system 150 kV/800 mA^4^, ^5^.

Abdominal CT angiography was performed in sternal recumbency, under general anesthesia after a transient period of hyperventilation to induce apnea and avoid motion artifacts. The animal was premedicated using butorphanol (0.3–0.4 mg/kg). Anesthesia was induced using propofol and followed by endotracheal intubation. Anesthesia was maintained using isoflurane gas and 100% oxygen. CT images were acquired using a multi-slice system with 16 or 64 slice detector^6^. Acquisition parameters used for the 16-multislice CT scanner were as follows: tube voltage 120 kV, reference tube current 200 mA, and pitch factor 0.8. Scan tube current was modulated by automatic exposure control (Care Dose, Siemens Medical Solutions, International). Image data sets were reconstructed using parameters of 200–300 mm field of view, 512 × 512 matrix, 1.5 mm slice thickness, and Br-20f reconstruction algorithm (window level 40 and window width 300) with filter back projection. For the 64-multislice CT scanner, acquisition parameters used were as follows: tube voltage 100 kV, reference tube current 260 mA, and pitch factor 0.6. Scan tube current was modulated by automatic exposure control (Care Dose, Siemens Medical Solutions, International). Image data sets were reconstructed using parameters of 100–200 mm field of view, 512 × 512 matrix, 0.75 mm slice thickness and Br-46 reconstruction algorithm (window level 88 and window width 404) with iterative reconstruction. Post-contrast images were acquired during the arterial, portal and venous phases after injection of 2 ml/kg of iodinated contrast medium (iohexol^7^ or iopromide^8^) with an automated injector.

## Results

### Animals

Nine animals, seven dogs and two cats (D1-7, C1-2; Table 1), corresponded to selection criteria. Breed was variable in the canine patients. Five dogs were intact females, one intact male and one neutered male. Age ranged from 6 months to 10 years (median 1.5 year-old). Both feline patients were neutered male domestic shorthair, aged 6 and 7 years.

**Table 1:** Signalement, history and final diagnosis of the nine animals with sclerosing encapsulating peritonitis.

**Patient**	**Age**	**Sex**	**Breed**	**History**	**Abdominal Radiographs**	**Abdominal Ultrasound**	**Abdominal CT**	**Final diagnosis and outcome**
D1	1 y	FI	Belgian malinois	Pregnancy	- Abnormal intestinal gas distribution and plication	- Free anechoic abdominal fluid - Hyperechoic intraabdominal fat - Mechanical ileus - Moderate abdominal lymphadenomegaly	N/A	Septic peritonitis *(Staphylococcus pseudintermedius)*
D2	2 y	FI	23kg crossed-breed	Pregnancy	N/A	- Free echogenic abdominal fluid focally surrounding some bowel loops - Focally hyperechoic intraabdominal fat - Small intestine hypoperistaltism with stick-like foreign body - Moderate mesenteric and medial iliac lymphadenomegaly	N/A	Intestinal perforating foreign body
D3	10 m	MN	Belgian malinois	Antescrotal castration	N/A	- Echogenic partially loculated abdominal fluid - Hyperechoic intraabdominal fat - Encased bowel loops - Small intestine hypoperistaltism with uncertain intraluminal foreign body - Severe jejunal and medial iliac lymphadenomegaly - Mass-like tissue along the abdominal wall	N/A	Necrotic pancreatitis, Septic peritonitis (*Pseudomonas putida)*
D4	2 y	FI	Beagle	Perforating intestinal foreign body and enterectomy	N/A	- Anechoic right periovarian fluid focally surrounding some bowel loops and the ovary - Small round and distorted spleen surrounded by a thin echogenic capsule	N/A	Ovariectomy, lateral flank approach
D5	6 m	FI	Leonberger	-	N/A	- Free anechoic abdominal fluid - Encased bowel loops - Small intestine hypoperistaltism with intraluminal grass ball - Severe jejunal lymphadenomegaly, moderate medial iliac lymphadenomegaly - Mass-like tissue along the abdominal wall	- Mesenteric fat stranding	Intestinal obstruction by grass ball, Neutrophilic enteritis and lymphangiectasis
D6	10 y	MI	Dachshund	Adopted	- Abdominal distention - Abnormal intestinal gas distribution and plication - Homogeneous peripheral fluid opacity	- Free abdominal fluid - Hyperechoic intraabdominal fat - Encased bowel loops - Small intestine hypoperistaltism with uncertain intraluminal foreign body	- Free abdominal fluid - Encased bowel loops - Mesenteric fat stranding - Equivocal thickening and increased contrast enhancement of the peritoneum	Scrotal leydigoma
D7	1 y	FI	German shepherd	-	N/A	- Echogenic loculated abdominal fluid - Hyperechoic intraabdominal fat - Encased bowel loops - Moderate jejunal lymphadenomegaly - Mesenteric echogenic mass and duodenal mass	- Loculated abdominal fluid - Encased bowel loops - Mesenteric fat stranding	Low grade duodenal leiomyosarcoma. *Recheck ultrasound exam on day 478 after initial presentation:* mechanical ileus
C1	6 y	MN	Domestic shorthair cat	-	N/A	- Echogenic loculated abdominal fluid - Hyperechoic intraabdominal fat - Encased bowel loops - Severe mesenteric lymphadenomegaly, especially jejunal lymph nodes	- Loculated abdominal fluid - Encased bowel loops - Mesenteric fat stranding - Equivocal thickening and increased contrast enhancement of the peritoneum	Cocci and bacilli on peritoneal capsule
C2	7 y	MN	Domestic shorthair cat	-	- Abdominal distention - Abnormal intestinal gas distribution and plication - Homogeneous peripheral fluid opacity	- Free echogenic abdominal fluid - Hyperechoic intraabdominal fat - Encased bowel loops - Moderate abdominal lymphadenomegaly - Mass and nodular images within cllumped mesenteric fat - Suspected pseudomembranous cystitis	N/A	Pseudomembranous cystitis, Septic peritonitis (*Listeria monocytogenes)*

*FI, intact female; MN, neutered male; MI, intact male; N/A, not applicable*.

### History and Clinical Findings

Two dogs had a recent surgery (uneventful antescrotal castration, and enterectomy) 2 weeks and 3 months before the diagnosis respectively. Two dogs had been pregnant, D1 with an unremarkable parturition 1 month before the diagnosis, and D2 with adhesions noticed by the referring veterinarian during C-section 10 months before. One dog had recently been adopted with an unknwon history (D6). The remaining four animals had an unremarkable clinical history.

Clinical signs at initial presentation included anorexia (6/9), lethargy (4/9), weight loss (4/9), abdominal distension (3/9), diarrhea (2/9), abdominal pain (1/9) and acute vomiting (1/9). D5 developed acute vomiting and diarrhea due to intestinal obstruction 1 month after the initial presentation and underwent enterotomy and ovariohysterectomy.

Bloodwork at initial presentation was available for 5/9 animals. Bloodwork was normal in one dog (D5). Four animals showed minor hematological or biochemical unspecific changes including mild to moderate neutrophilia (D1, D3, C1 and C2), mild lymphocytosis and monocytosis (C1), mild hyper- (D1, C2) or hypoglycemia (C1), pre-renal azotemia (C2), and mild hypoproteinemia and increase in alkaline phosphatase level (D3).

### Radiographic and Ultrasonographic Findings

Two dogs (D1, D6) and one cat (C2) had abdominal radiographic examination and all animals underwent an abdominal ultrasonographic examination.

**Figure 1:**
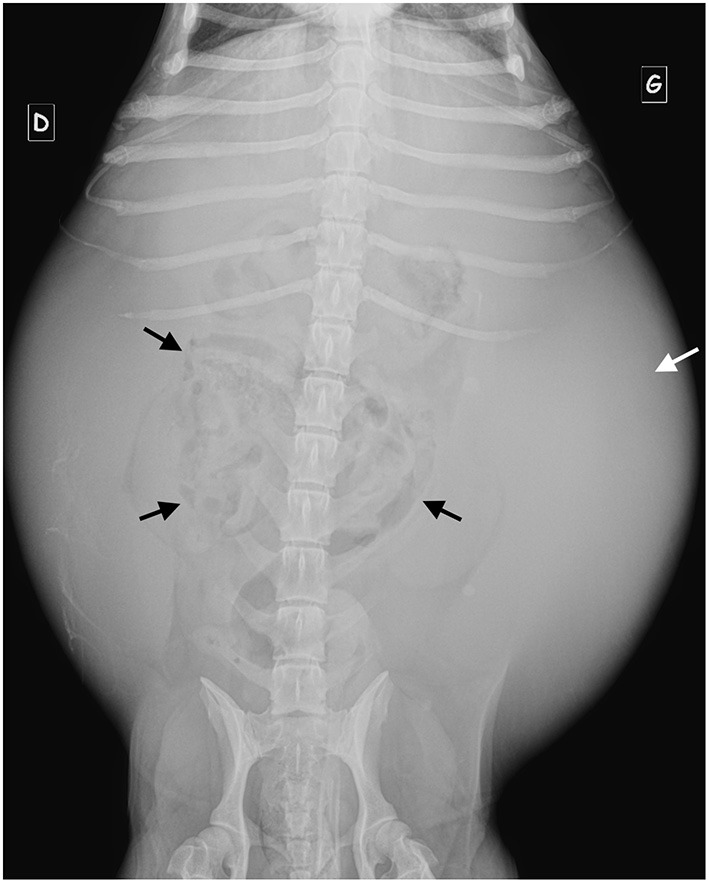
Ventro-dorsal radiograph of D6. Note the severely increased abdominal volume, the central gathering of the small bowel loops (black arrows) with fluid opacity at the periphery of the peritoneal cavity (white arrow).

Radiographs showed increased abdominal volume in 2/3 animals (Figure 1). Reduced serosal detail and an abnormal geometric, plicated shape of intestinal gas content were present in all three animals, with bowel loops stacked in the central abdomen and surrounded by a large amount of fluid (2/3). These radiographic findings were considered suggestive of either the presence of a linear foreign body or multiple serosal adhesions.

**Figure 2:**
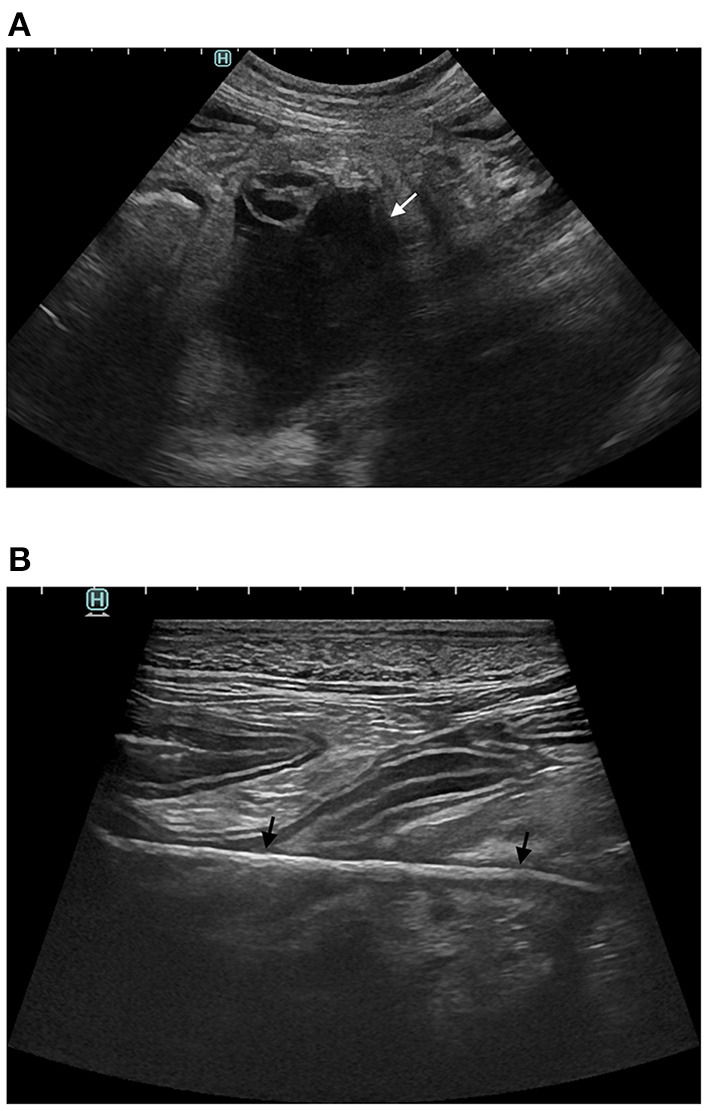
Abdominal ultrasound of D2. **(A)** Echoic fluid filled cavity (white arrow) along a small bowel loop **(B)** Perforating intestinal stick-like foreign body (black arrows).

**Figure 3:**
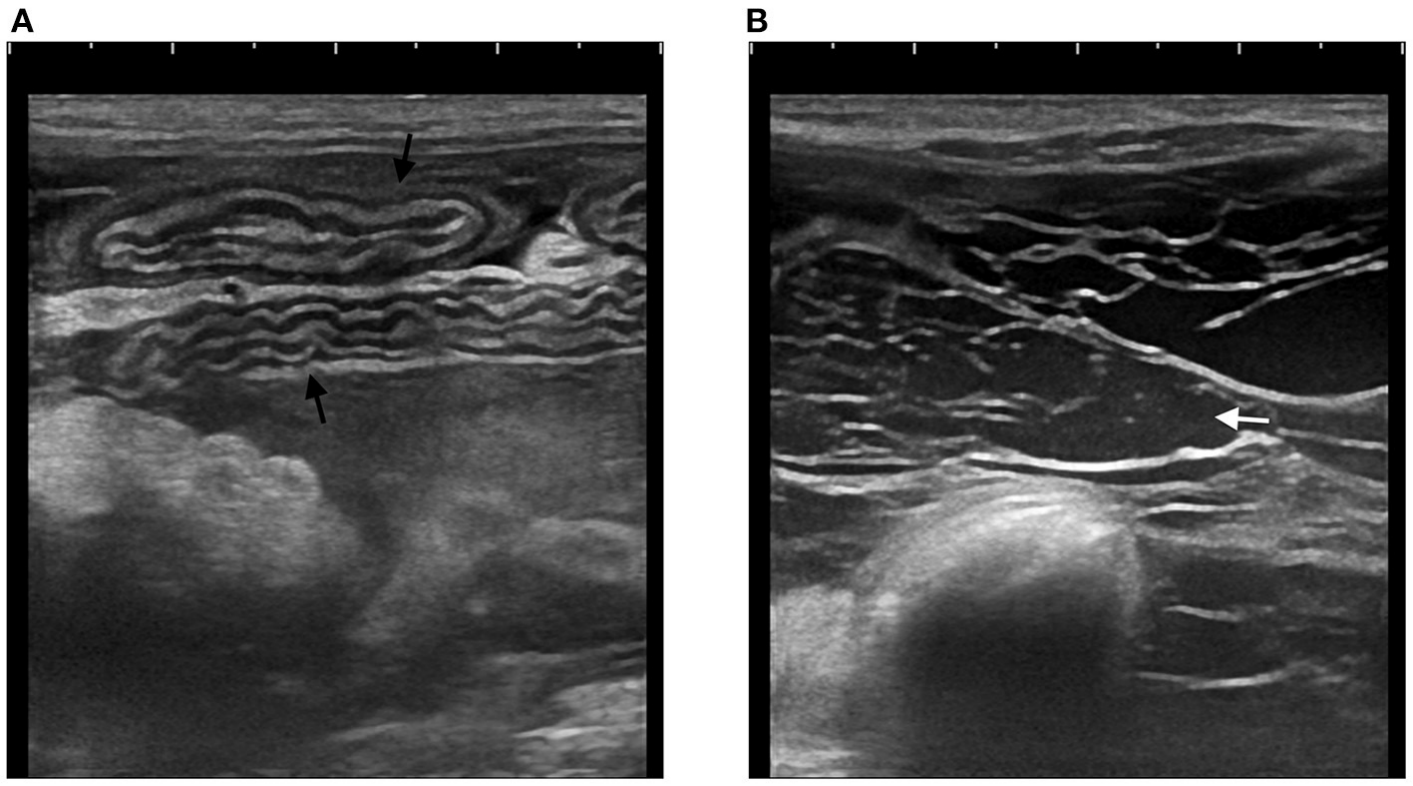
Abdominal ultrasound of C1. **(A)** Multiple segments of corrugated small intestine (black arrows) **(B)** Echoic peritoneal fluid (white arrow) with multiple hyperechoic septations.

Sonographically free fluid was visible in all animals, either distributed in the entire peritoneal cavity (7/9) or focally collected (2/9). Peritoneal effusion was echogenic (6/9) and loculated or partially loculated by thin echogenic septations (3/9), suggesting an inflammatory process. Bowel loops had a plicated (7/9) and/or corrugated (7/9) appearance in all animals suggesting the occurrence of adhesions and bowel inflammation (Figure 2). In 6/9 animals the clumped bowel loops were encased and peritoneal fluid relegated separately in a different area of the abdominal cavity. In the two animals where peritoneal effusion was collected focally, the fluid was in close contact with a corrugated small bowel loop (in the area where a wooden foreign body was found perforating the bowel wall in D2 and in the right ovarian area concomitant with a periovarian effusion in D4) (Figure 3). Small bowel motility was considered reduced in 5/9 animals (D2 with a stick-like foreign body, D5 with grass ball, D1, D3 and D6 with uncertain intraluminal small foreign materials) and mechanical ileus occurred in 2/9 animals (D1 and D7, respectively on day 1 and day 478 after initial presentation). Intraabdominal fat was hyperechoic in 7/9 animals (focally in D2 surrounding the stick-like foreign body), suggesting steatitis. Moderate or severe abdominal visceral and/or parietal lymphadenomegaly was seen in 8/9 animals with the jejunal lymph nodes being predominantly involved in 4/9 animals.

Additional ultrasound findings were present in 5/9 animals. Two dogs (D3, D5) had thickened heterogeneous tissue mimicking a mass adherent to the abdominal wall at the site of laparotomy. D7 had an ovoid mesenteric echogenic mass unrelated to any abdominal organ and a small eccentric duodenal mass originating from the muscular layer. D4 had a small round distorted / folded spleen, surrounded by a thin echoigenic capsule. C2 had mass and nodular images (respectively ≥3 cm and <3 cm) within the clumped mesenteric fat and images of the bladder consistent with pseudomembranous cystitis.

### CT Findings

Four animals underwent an abdominal CT (including contrast examination) (D5, D6, D7 and C1) for surgical planning.

**Figure 4:**
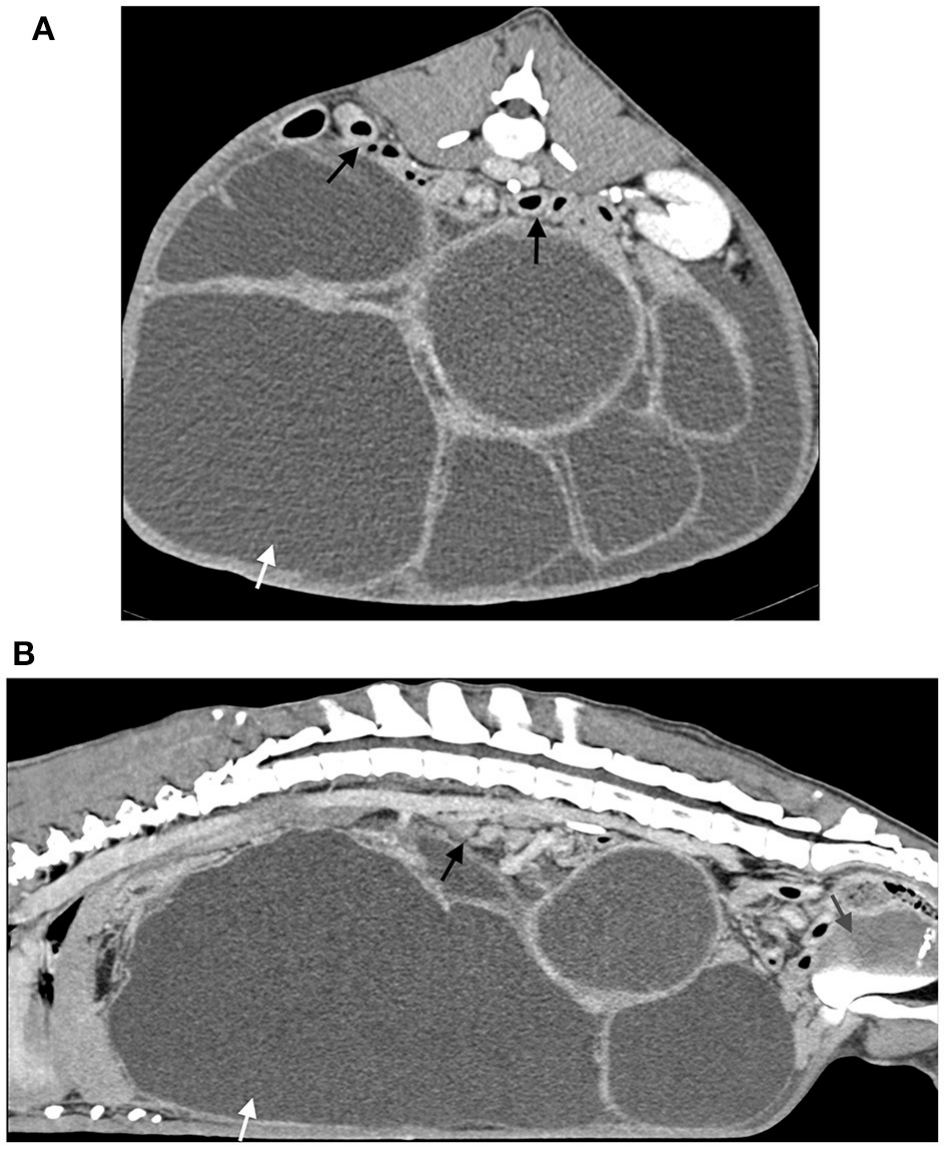
Post-contrast CT acquisition of the abdomen of D7. **(A)** Transverse image, soft tissue window, at the level of the left kidney **(B)** Sagittal multiplanar reconstruction image, soft tissue window. A large amount of peritoneal fluid (white arrow) loculated by multiple hyperattenuating septations and dorsal gathering of the intestines is present (black arrows). Note also the mass effect of the fluid filled cavities and the secondary caudal displacement of the urinary bladder (gray arrow).

**Figure 5:**
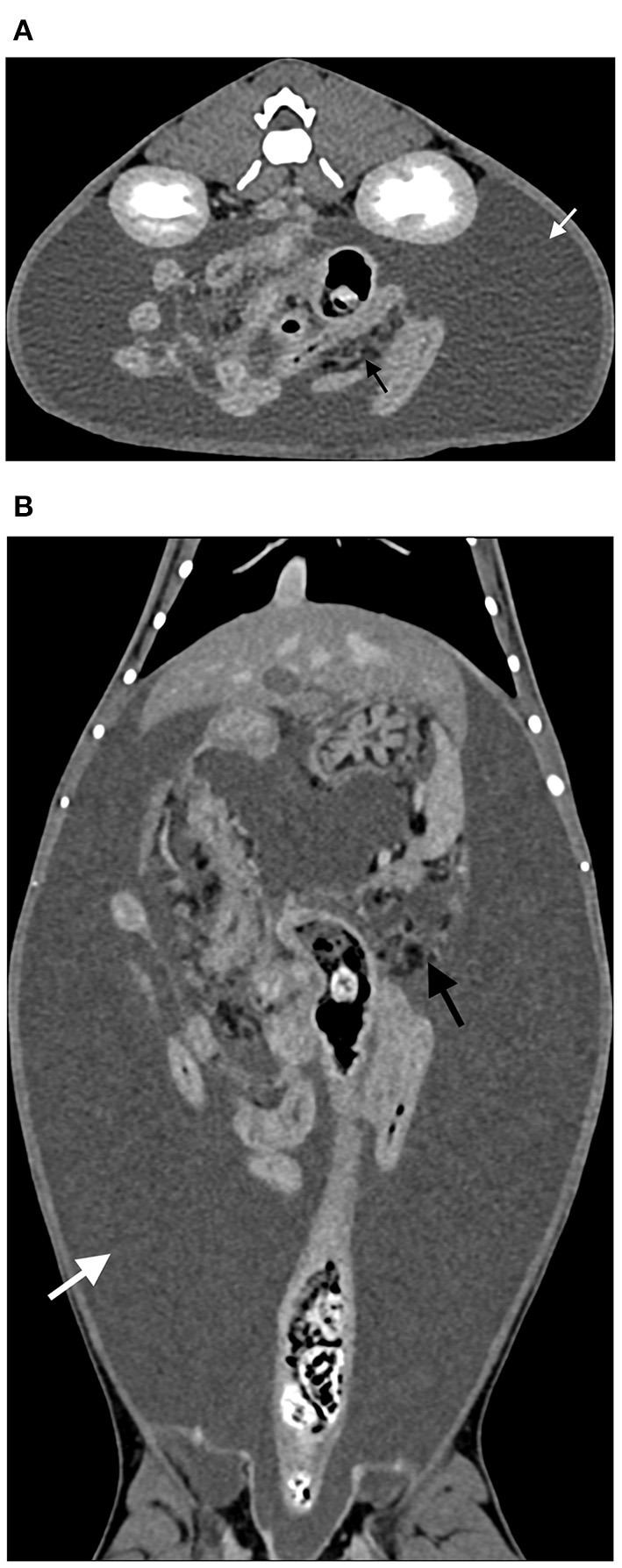
Late post-contrast CT acquisition of the abdomen of C1 (same case as Figure 3). **(A)** Transverse image, soft tissue window, at the level of the kidneys **(B)** Dorsal multiplanar reconstruction image, soft tissue window, at the level of the descending colon. There is central gathering of the intestines and a large amount of surrounding peritoneal fluid (white arrow). Fat stranding is also visible (black arrow).

Abdominal CT examinations confirmed the ultrasound findings in 4/4 animals. Free or loculated abdominal fluid (3–15 Hounsfield units) and encased bowel loops were confirmed in all three animals where this distribution had already been suspected based on radiographs and ultrasound (Figures 4, 5). CT images also showed mesenteric fat stranding characterized by an increased linear or ill-defined attenuation in 4/4 animals. Extraluminal adhesions were suspected in 3/4 animals and in 2/4 animals an equivocal thickening and increased contrast enhancement of the peritoneum was noticed.

### Surgical and Histopathological Findings

**Figure 6:**
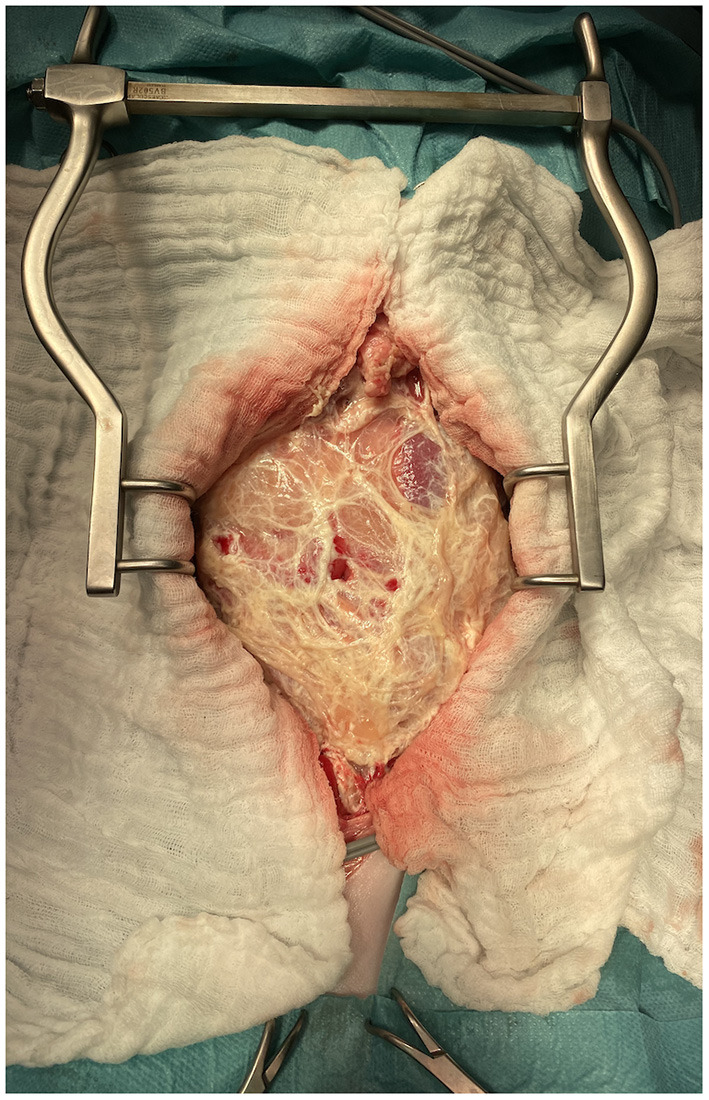
Intraoperative picture of the laparotomy of C2 after abdominal wall incision. Note the thick fibrous membrane encapsulating the abdominal viscera.

All nine animals underwent exploratory laparotomy. Surgery confirmed the presence of a thick capsule around the abdominal viscera (6/9), multiple serosal adhesions between small bowel loops (6/9) or between small bowel loops and ovary (1/9) or abdominal wall (2/9) (Figure 6). In the dog with the stick foreign body (D2) abdominal abscesses were present. Pancreatic necrosis was found in D3. Neutrophilic enteritis with associated mild lymphangiectasis was confirmed by histopathology in D5. Neoplasia was confirmed in C2, D7 and D6, with respectively carcinomatosis associated to marked fibrosis, a low grade duodenal leiomyosarcoma and a scrotal leydigoma. Analysis of peritoneal fluid was performed in 5/9 animals and revealed a septic exudate in D1 (*Staphylococcus pseudintermedius*) and C2 (*Listeria monocytogenes*) and a non-septic modified transudate in D6, D7 and C1. Additionally, prior to referral, D6 had a positive bacterial culture (*Pseudomonas putida*) on peritoneal effusion. Fibrinous adhesions were submitted for histopathological analysis in 4/9 animals and confirmed either acute (C1 and D1) or chronic (C2 and D6) fibrinous neutrophilic inflammation. Cocci and bacilli were also present on the histopathological analysis of the fibrinous material in C1. Although most animals survived several months to years after initial admission (more than 3 to 20 months post diagnosis for 6/9 animals), 3/9 animals, including both cats, died or were euthanised within 7 days due to complications or lack of clinical improvement post-surgery.

## Discussion

This case series describes imaging findings in seven dogs and two cats with confirmed SEP. Most animals included were either juveniles or young adults (<3 years old). Previous reports show a wide age distribution demonstrating that juvenile (9, 12, 14, 16) as well as mature (7, 8, 10, 11, 13, 15) animals may be affected. Intact females were overrepresented (5/9) in contrary to previous reports which do not suggest any sex or neutered status influence (7, 8, 10–12, 15, 16).

SEP has been referred to be *idiopathic* or *secondary* depending on previous conditions that may be identified as potential trigger of peritoneal inflammation (1, 2). Foreign bodies and necrotic neoplastic masses have been reported as potential initiating causes of abdominal inflammation in SEP (8, 10, 16). In the present case series one dog had undergone a previous abdominal surgery, one dog had a perforating foreign body and two had been pregnant prior or at the time of the diagnosis of SEP. Neoplasia was found in three other dogs (one carcinomatosis, one duodenal leyomyosarcoma and one scrotal leydigoma) and necrotic pancreatitis was found in one. These associated medical conditions may have been potential triggers for peritoneal inflammation. One dog had been castrated prior to the diagnosis of SEP (D6) and an ascending infection or systemic inflammation may have occurred.

The diagnostic imaging abnormalities in this case series predominantly involved the intestinal tract and peritoneal space. Intestinal findings most commonly involved plication and corrugation (consistent with the presence of adhesions in the absence of a foreign body) associated with peritoneal effusion and lymphadenomegaly. Sonographically, peritoneal fluid was often echogenic (6/9) and frequently associated with echogenic septae suggesting an inflammatory cause. This hypothesis is in accordance with previous descriptions in case reports (7, 8, 11, 12, 15, 16) and imaging text-books (18, 19). Thickening of the serosal surface and parietal peritoneum is also a sonographic finding described in textbooks (18, 19) but was not clearly seen in this retrospective case series.

Adhesions around and between small intestinal loops sometimes with an encapsulated visceral mass (6/9) was a common surgically finding. This characteristic encapsulation was seen as a gathering of small bowel loops in the central abdomen surrounded by fluid on radiographic, CT and ultrasonographic examinations of respectively 2/3, 3/4 and 5/9 animals. In two animals a mechanical ileus developed after the first onset of symptoms and this is similar to what is described in humans where mechanical ileus is reported as a consequence of adhesions and impaired bowel motility (1, 2).

Four cases had undergone a CT examination. Consistently with findings reported in literature (1, 2, 16), thickening of the serosa or adhesions and peritoneal fat stranding were noticed on CT images. Fat stranding refers to an abnormal increased attenuation in fat because of edema and engorgement of lymphatics (20). In humans, fat stranding has been found to be very sensitive for peritoneal inflammation, being a key CT feature to differentiate uncomplicated from complicated appendicitis. Whereas mild inflammation causes a subtle hazy increased attenuation of the fat (ground-glass like), severe inflammation produces a reticular pattern (21, 22). In the animals where CT was performed linear/reticular fat stranding was present either in the entire peritoneal cavity or focally around the abnormal bowel loops. At contrast CT, two animals had serosal enhancement, and this is consistent with reports in human patients with SEP (2).

While the entire peritoneal cavity was involved in the majority of animals, two cases had focal ultrasound abnormalities with peritoneal effusion and hyperechoic fat being localized around an abnormal corrugated small bowel loop. These animals may either represent an earlier stage of the disease or simply better show the undergoing focal triggering process. However, if previous surgery is reported, animals with focal implication of the abdominal cavity may be difficult or impossible to distinguish from animals suffering of post-operative adhesions and not necessarily progressing to SEP.

The main limitations of this study are its retrospective nature and the small sample size. The complementary examinations were at the discretion of the clinician after consideration of the animal clinical presentation and owner budget. In particular, 5/9 animals did not have CT examination, 6/9 did not have abdominal radiographs and 5/9 did not have histopathological analysis of the peritoneal adhesions and membranes. However, because of the rarity of the disease, especially in cats (5, 6, 14), and its life threatening character a small case series assembling a detailed description of the most common imaging findings of SEP is considered useful to develop a better pre-surgical diagnosis in veterinary patients.

## Conclusions

This small case series confirms findings previously reported in isolated SEP cases. As clinical presentation may be nonspecific, diagnostic imaging should be considered to exclude other disorders. Imaging findings of SEP in dogs and cats include abnormal shape and distribution of small bowel loops on radiographs, plicated and/or corrugated small bowel loops with echogenic loculated peritoneal effusion on ultrasound and fat stranding on CT.

## Data Availability Statement

The original contributions presented in the study are included in the article/supplementary material, further inquiries can be directed to the corresponding author.

## Ethics Statement

The animal study was reviewed and approved by Comission d'Ethique Animale, Université de Liège. Written informed consent for participation was not obtained from the owners because this is a retrospective study using clinical datas. The Ethical Committee retrospectively approved the study (File number 22-2459) on the 11th of May 2022.

## Author Contributions

BG and VB contributed to conception and design of the study. BG and CP gathered database. BG organized the database and wrote the first draft of the manuscript. VB contributed to manuscript revision and wrote sections of the manuscript. All authors contributed to manuscript revision, read, and approved submitted version.

## Conflict of Interest

The authors declare that the research was conducted in the absence of any commercial or financial relationships that could be construed as a potential conflict of interest.

## Publisher's Note

All claims expressed in this article are solely those of the authors and do not necessarily represent those of their affiliated organizations, or those of the publisher, the editors and the reviewers. Any product that may be evaluated in this article, or claim that may be made by its manufacturer, is not guaranteed or endorsed by the publisher.

## Footnotes

^1^Aloka 3500 Prosound and Aloka Prosound SSD-Alpha10, Aloka Co. Ltd., Tokyo, Japan.

^2^Arietta 850 SE, Hitachi-Fujifilm, Mechelen, Belgium.

^3^Canon Aplio a, Sint-Stevens-Woluwe, Belgium.

^4^Agfa Musica DR 14s and CR35x, Mortsel, Belgium.

^5^X-ray table General Electric CGR, Diegem, Belgium.

^6^Siemens, Somatom 16 and Somatom Confidence 64, Erlangen, Germany.

^7^Omnipaque 300, General Electric Healthcare, Diegem, Belgium.

^8^Ultravist 300, Bayer Pharma, Diegem, Belgium.

## Abbreviations

CT: Computed tomography
SEP: sclerosing encapsulating peritonitis
FI: intact female
MN: neutered male
MI: intact male
N/A: not applicable.
